# Double-Modal Locomotion of a Hydrogel Ultra-Soft Magnetic Miniature Robot with Switchable Forms

**DOI:** 10.34133/cbsystems.0077

**Published:** 2024-01-08

**Authors:** Shihao Zhong, Zhengyuan Xin, Yaozhen Hou, Yang Li, Hen-Wei Huang, Tao Sun, Qing Shi, Huaping Wang

**Affiliations:** ^1^ Intelligent Robotics Institute, School of Mechatronical Engineering, Beijing Institute of Technology, Beijing 100081, China.; ^2^ Peking University First Hospital, Beijing 100034, China.; ^3^ Laboratory for Translational Engineering, Harvard Medical School, Cambridge, MA 02139, USA.; ^4^ Beijing Advanced Innovation Center for Intelligent Robots and Systems, Beijing Institute of Technology, Beijing 100081, China.; ^5^ Key Laboratory of Biomimetic Robots and Systems (Beijing Institute of Technology), Ministry of Education, Beijing 100081, China.

## Abstract

Flexible miniature robots are expected to enter difficult-to-reach areas in vivo to carry out targeted operations, attracting widespread attention. However, it is challenging for the existing soft miniature robots to substantially alter their stable shape once the structure is designed. This limitation leads to a fixed motion mode, which subsequently restricts their operating environment. In this study, we designed a biocompatible flexible miniature robot with a variable stable form that is capable of adapting to complex terrain environments through multiple movement modes. Inspired by the reversible stretching reaction of alginate saline gel stimulated by changes in environmental ion concentration, we manufactured a morphologically changeable super-soft hydrogel miniature robot body. According to the stretch and contraction shapes of the flexible hydrogel miniature robot, we designed magnetic fields for swing and rolling motion modes to realize multi-shape movement. The experimental results demonstrate that the deflection angle of the designed flexible miniature robot is reversible and can reach a maximum of 180°. The flexible miniature robot can complete forward swinging in the bar stretch state and tumbling motion in the spherical state. We anticipate that flexible hydrogel miniature robots with multiple morphologies and multimodal motion have great potential for biomedical applications in complex, unstructured, and enclosed living environments.

## Introduction

Miniature robots are highly anticipated to realize biomedical applications in confined and narrow areas within living bodies [1–4], such as disease diagnosis [5], minimally invasive surgery [6], immunotherapy [7], and drug delivery [8]. However, due to their small size, miniature robots are difficult to carry onboard energy-powered devices and require external physical fields to provide wireless driving force [4]. Common external driving sources for miniature robots include light [9], sound [10], electric [11], and magnetic fields [12]. Since low-intensity magnetic fields can penetrate biological tissues without causing damage and provide a driving force for miniature robots in confined spaces within living bodies, they are particularly well-suited for in vivo applications [13–17]. Magnetic miniature robots can be classified into rigid and flexible structures. Compared to rigid structures, flexible bodies are safer due to their similar hardness to biological tissues, which reduces the risk of tissue damage. Additionally, flexible miniature robots have a higher degree of freedom of movement and can adapt more flexibly to complex internal environments within living bodies [18–22]. Therefore, it is necessary to develop bio-compatible, magnetically driven ultra-soft miniature robots.

In recent years, there have been reports on flexible miniature robots featuring diverse structures and driving modes. Huang et al. [23] designed a miniature robot with a flexible spiral tail that can rotate at the same frequency as an external rotating magnetic field to generate axial displacement. Driven by an oscillating magnetic field with asymmetric reverse changes, the bionic scallop-shaped flexible robot designed by Qiu et al. [24] and the starfish-shaped flexible robot designed by Zheng et al. [25] can perform opening and closing motions to generate displacement. The hinge structure designed by Li et al. [26] and the bionic fish structure designed by Xu et al. [27] can be driven by a symmetrical oscillating magnetic field. Ze et al. [28] designed an origami mechanism with reversible deformation that can generate displacement through magnetic field excitation. Huang et al. [29] designed a quadruped soft miniature robot driven by rotating or conical magnetic fields. Due to the inability to markedly modify the stable shape of existing flexible miniature robots once they are designed, their motion modes remain fixed, leading to limited operating environments. Therefore, to improve their adaptability to different environments, it is necessary to study flexible miniature robots with variable stable forms and multiple motion modes.

In this study, we prepared a biocompatible ultra-soft miniature robot with alterable morphic using electrodeposition of alginate brine gel and demonstrated its multimodal motion control. Based on the properties that alginate saline gel can undergo reversible stretching reactions in response to changes in environmental ion concentration, a deformable miniature flexible robot body of ultra-soft hydrogel was constructed. We designed the corresponding motion mode and driving magnetic field according to the stretch and contraction shape of the hydrogel flexible miniature robot to realize the multi-shape motion. The flexible miniature robot is capable of performing forward swinging in the bar stretch state and tumbling motion in the spherical state. Furthermore, the miniature robot possesses the ability to perform in situ steering, enabling it to navigate through extremely narrow slits during both entry and exit. We believe that flexible hydrogel miniature robots with multiple morphologies and multimodal motion have great potential for biomedical applications in complex, unstructured, and enclosed living environments.

## Materials and Methods

### Design and fabrication of the flexible miniature robot

**Fig. 1.**
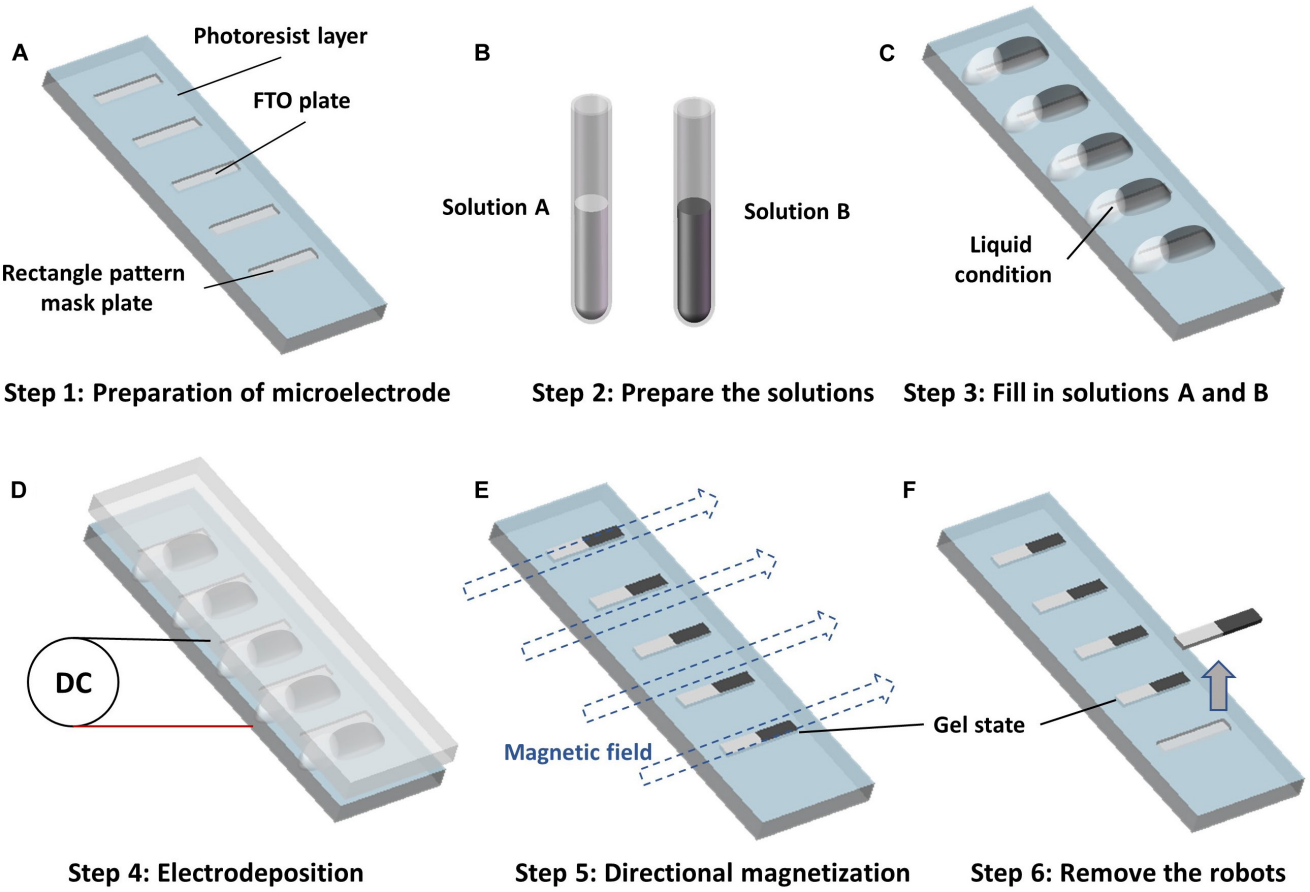
A conceptual overview of the fabrication of the flexible miniature robot. (A) The rectangle pattern mask plate. (B) Solutions A (containing sodium alginate and CaCO_3_) and B (containing solution A and NdFeB particles). (C) A flexible miniature robot equipped with a magnetic head and a non-magnetic tail. (D) Schematic diagram of electrodeposition manufacturing. (E) Schematic diagram of axial magnetization. (F) The preparative flexible miniature robot.

The rectangular-shaped alginate hydrogel flexible miniature robot has dimensions in a scale of 100:5:2, representing the major axis length, minor axis length, and thickness, respectively. Comprising a magnetic head and non-magnetic tail, this hydrogel flexible miniature robot was crafted via a microelectrode-based method using soft alginate hydrogel. As depicted in Fig. 1, our fabrication process began by processing photoresist onto an SnO_2_:F (FTO) plate to create the desired rectangle pattern. Since the photoresist layer conducts no electricity, only electrodes with the desired pattern generate electric fields.

Next, we dripped deposition solutions A (containing sodium alginate and CaCO_3_) and B (containing solution A and NdFeB microparticles), respectively, onto the rectangular FTO plate as shown in Fig. 1C. With the second FTO plate covering this setup, the gap between both plates was filled with the deposition solution, and a constant current was passed into the FTO plates to initiate electrolysis, as shown in Fig. 1D. The electrolysis process generated H^+^ ions on the anode plate surface, reducing pH rapidly and stimulating the electrodeposition process. H^+^ ions then reacted with CaCO_3_ particles to produce CO_2_ and Ca^2+^ ions that, in turn, reacted with sodium alginate to achieve Ca-alginate gelation.

Following electrodeposition, we placed the resulting alginate hydrogel flexible miniature robot into a 700-mT magnetic field (see Fig. 1E). The internal magnetic particles were magnetized through an external magnetic field application, and we removed the robot after the procedure of magnetization was completed.

### Reversible deformation mechanism of the flexible miniature robot

**Fig. 2.**
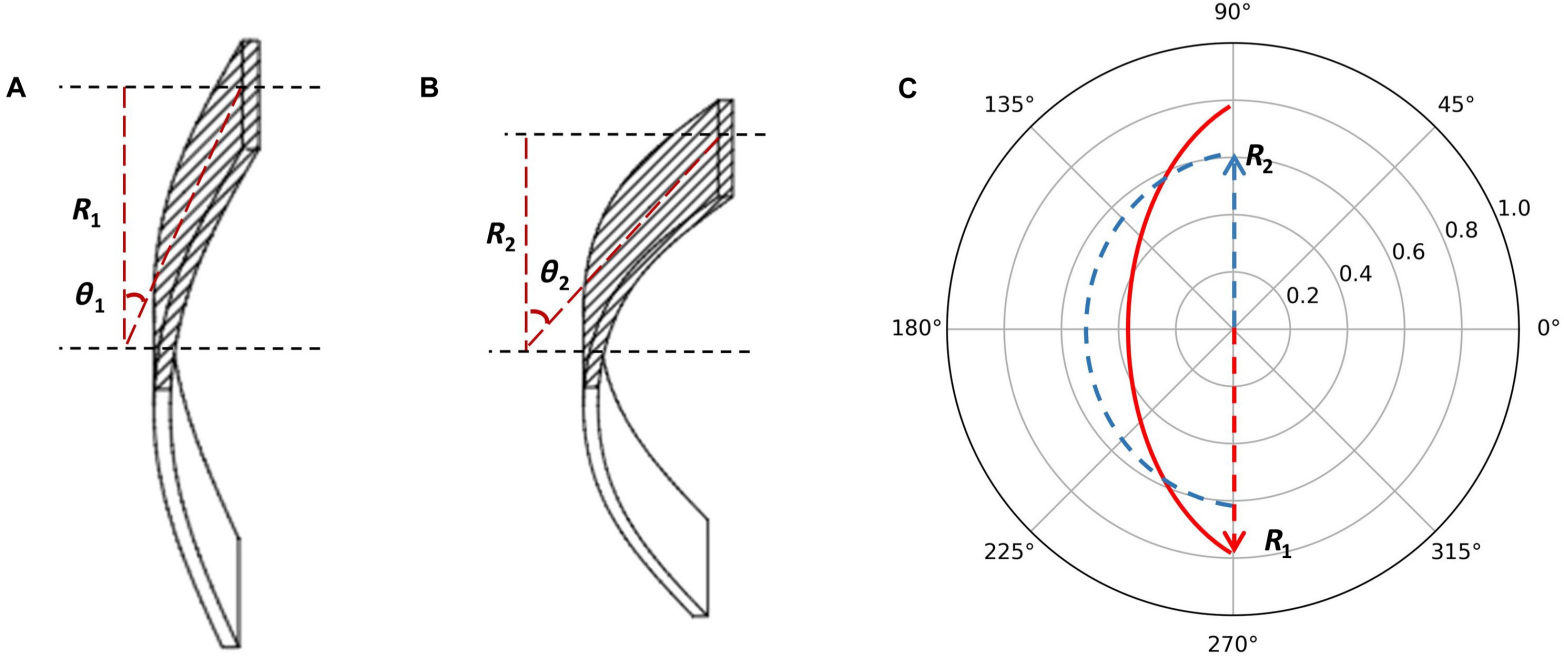
The flexible miniature robot structure parameter definition. *θ* indicates the deformation angle and *R* stands for morphological radius. (A) The deformation at pH *p*_1_ is characterized by an angular displacement denoted as *θ*_1_. (B) The deformation at pH *p*_2_ is characterized by an angular displacement denoted as *θ*_2_. (*P*_1 _> *P*_2_, *θ*_1 _< *θ*_2_). (C) Deformation scheme.

During the electrodeposition process in fabricating the flexible miniature robot, the edge effect of the microelectrode caused the generation of a heterogeneous electric field that led to uneven gel network density. Such a heterogeneous network can result in the reversible deformation of the flexible miniature robot. Figure 2 shows the flexible miniature robot ontology parameter setting. In this study, we assume that the contraction of the square bar of the flexible miniature robot is symmetric. *θ* indicates the deformation angle, meaning the degree to which the flexible miniature robot is shifted from the linear state and *R* stands for morphological radius (half the vertical length of the current robot). In an acidic liquid environment (1 < pH < 3), H^+^ ions react with calcium carbonate CaCO_3_ to produce Ca^2+^ ions, which combine with alginate through ionic crosslinking to form alginate hydrogels. This process coordinates Ca^2+^ ions with 4-carboxyl groups, resulting in an egg-box arrangement that causes the gel network to shrink. The uneven shrinkage of the gel network deforms the flexible miniature robot.

Conversely, alginate hydrogels exhibit an inverse phenomenon, where functional acid groups such as −COO^−^ undergo protonation at higher pH values (pH > 5) or react with monovalent cations. This results in water absorption and triggers a loose gel network, causing the flexible miniature robot to return to its original shape.

### Magnetic actuation and locomotion pattern

The flexible miniature robot is subjected to magnetic force *F*_*m*_ and torque *T*_*m*_ in a magnetic field [30].

$$T_{m}=m\times B$$ (1)

$$F_{m}=\left(m•\nabla \right)B$$ (2)

where ***m*** is the magnetic moment vector of the miniature robot, ***B*** is the external magnetic field vector, and ∇ is the gradient operator.

The external magnetic field is controlled by currents input through the electromagnetic coils. In the working area, the magnetic fields generated by the multi-stage coils can be linearly superimposed.

$$B\left(p\right)=\sum_{i=1}^{n}B_{0}I_{i}$$ (3)

where ***p*** is the position vector, *n* is the number of solenoid coils, *I*_*i*_ is the current through the electromagnet of the *i*th stage, and *B*_0_ is each coil at the center of the unit input current magnetic induction at the center.

The relationship between the spatial magnetic field and the current at each level can be written as

$$\left[\begin{array}{c} B_{x}\\ B_{y}\\ B_{z} \end{array}\right]=B_{0}D\left[\begin{array}{c} I_{1}\\ \overset{\;}{\vdots }\\ I_{n} \end{array}\right]$$ (4)

where ***D*** is the directional matrix of the multistage coil, which has full rank but is not a square matrix. In order to calculate the required current, the most direct and efficient way is to solve the pseudo-inverse of the direction matrix.

$$I=B_{0}^{-1}D^{†}B$$ (5)

**Fig. 3.**
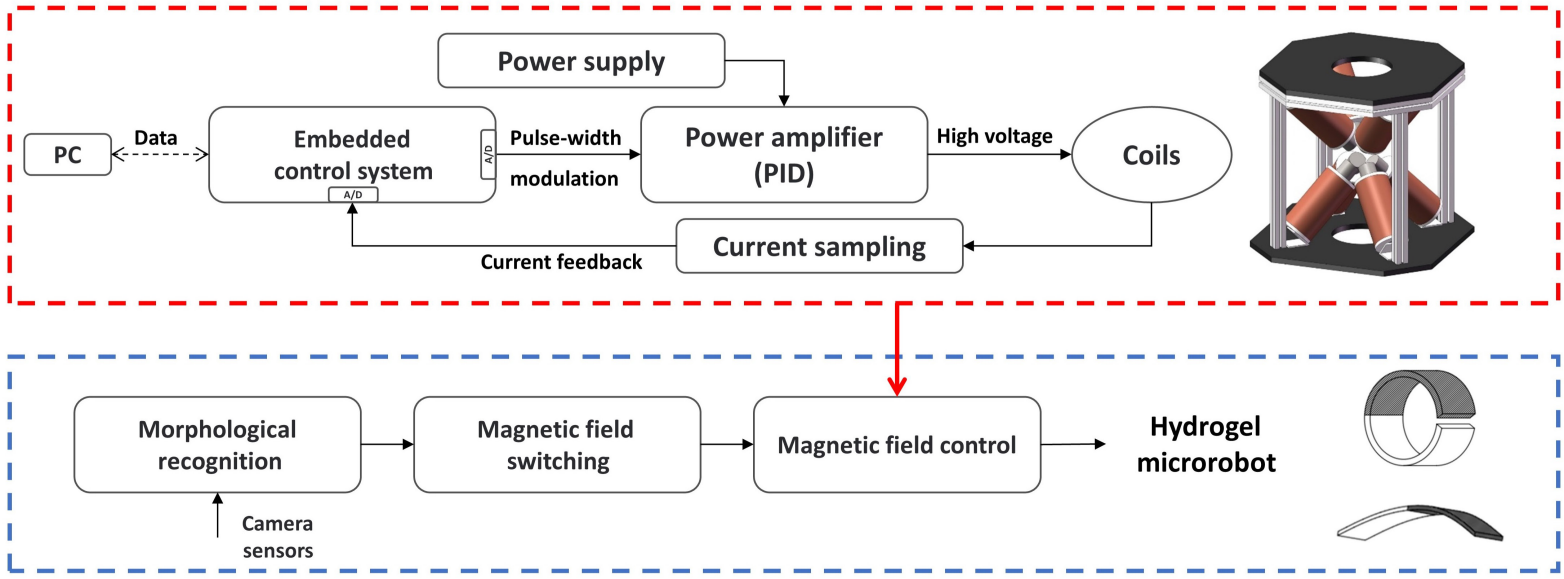
Schematic diagram of the electromagnetic drive for flexible miniature robots.

In order to accurately output the desired magnetic field, a feedback control system is constructed in this study. The control block diagram is shown in Fig. 3. The current driving magnetic field type is determined by the image feedback of the current shape of the flexible microrobot. Magnetic field accuracy is indirectly controlled by current feedback control. Within the magnetic field control system, the upper computer transmits the control signal for the magnetic field output current to the lower computer, employing a calculation relationship between the magnetic field and the current. The lower machine then generates the desired current utilizing the PID control algorithm.

**Fig. 4.**
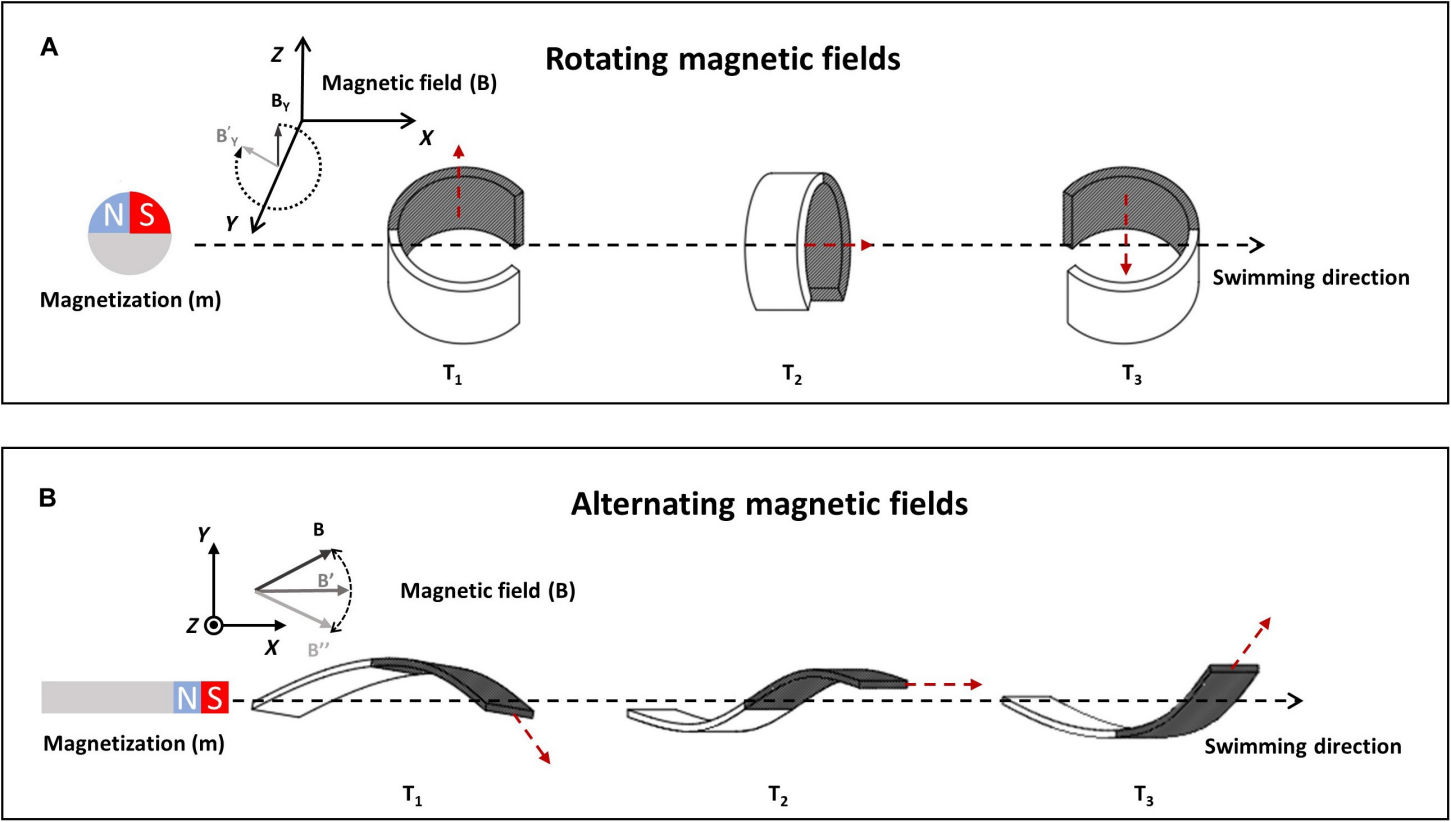
Schematic diagram of 2 motion modes and their magnetic driving modes. (A) The rolling motion driven by the external rotating magnetic field. (B) The pendulum motion driven by the external oscillating magnetic field. The red arrow points to the magnetic field.

1. Rolling motion: When the flexible miniature robot is deformed, it takes on a spheroid shape and can be driven to roll by a rotating field, as shown in Fig. 4A. The magnetic component of the flexible miniature robot can rotate synchronously with the external rotating magnetic field. The direction and speed of the flexible miniature robot can be altered by adjusting the direction and frequency of the rotating magnetic field. The rotating magnetic field can be given by

$$B_{N}=RB_{0}$$ (6)

$$R=\left[\begin{array}{ccc} C+A_{x}^{2}\left(1-C\right) & A_{x}A_{y}\left(1-C\right)-A_{z}S & A_{x}A_{z}\left(1-C\right)+A_{y}S\\ A_{x}A_{y}\left(1-C\right)+A_{z}S & C+A_{y}^{2}\left(1-C\right) & A_{y}A_{z}\left(1-C\right)-A_{x}S\\ A_{x}A_{z}\left(1-C\right)-A_{y}S & A_{y}A_{z}\left(1-C\right)+A_{x}S & C+A_{z}^{2}\left(1-C\right) \end{array}\right]$$

where *C* =  cos*φ*, *S* =  sin*φ*, *A* is the unit vector of the rotation axis, and *φ* is the rotation angle. ***B***_0_ is the initial magnetic field vector.

2. Pendulum motion: When the flexible miniature robot does not deform, it can imitate the forward swimming motion of a fish by oscillating actuation fields, as shown in Fig. 4B. The magnetic part vibrates at the same frequency as the applied oscillating magnetic field, while the non-magnetic part is pulled and swings with lag. The direction and speed of the flexible miniature robot can be altered by adjusting the direction and frequency of the oscillating magnetic field. The oscillating magnetic field can be given by

$$\begin{array}{c} B\left(f\;,t\right)=B\left[\text{sin}\;\alpha \;\text{sin}\left(2\pi \;\mathrm{ft}\right)n+\text{cos}\;\alpha u\right] \end{array}$$ (7)

where ***n*** and ***u*** are the orthogonal vectors in the *X*–*Y* plane. *α*, *f*, and *B* represent an oscillating angle, an oscillating frequency, and the amplitude of the oscillating magnetic field, respectively.

## Results

### Experimental setup

**Fig. 5.**
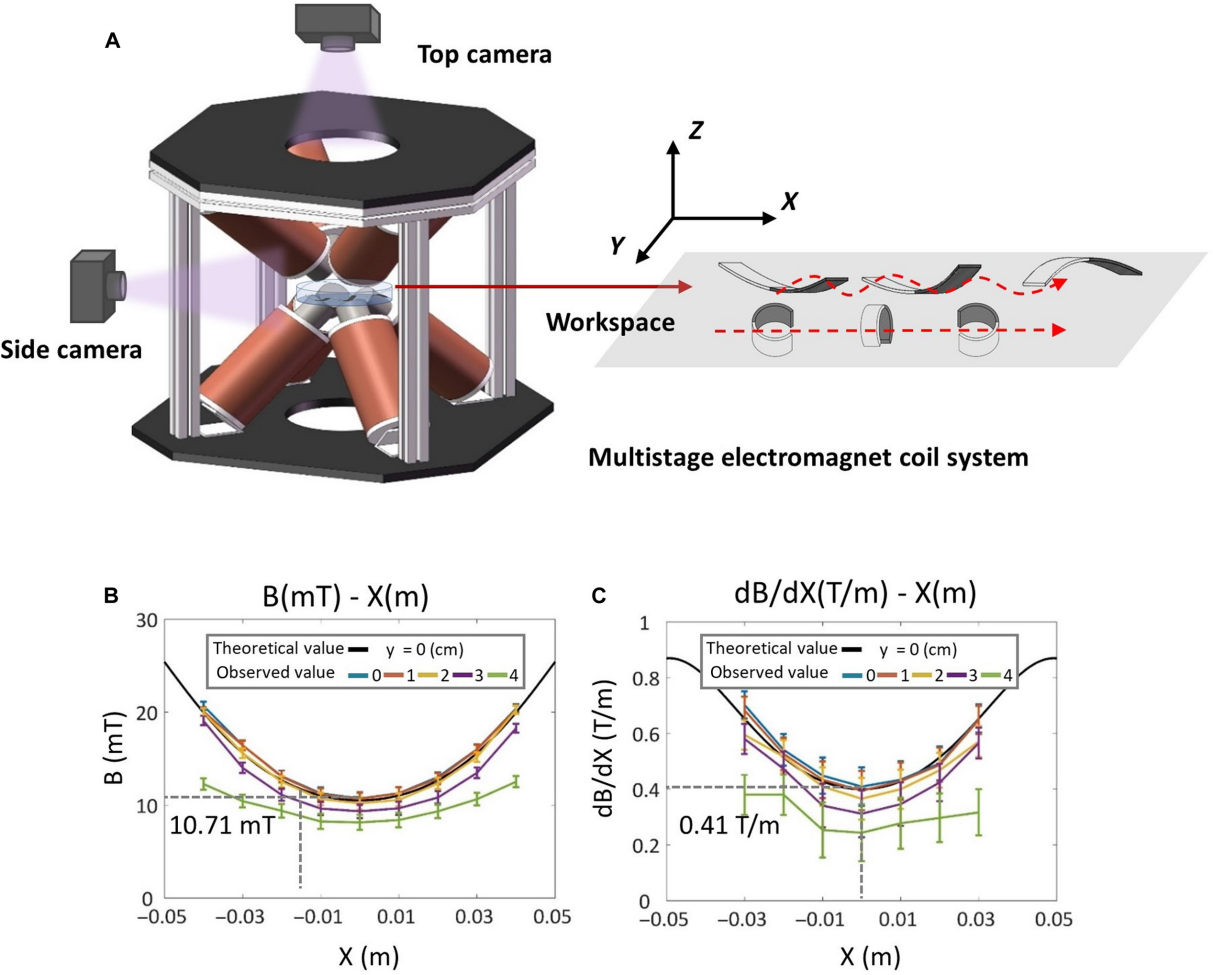
Schematic diagram of the experimental environment. (A) The electromagnetic drive system includes the 8-stage electromagnet coil system and the binocular orthogonal microscopic observation system. (B) Magnetic distribution. (C) Graded distribution.

In this study, the motion of the flexible miniature robot is actuated by a self-made multistage electromagnet coil system, in which the octet coil is distributed along the vertices of a cuboid, as shown in Fig. 5. Each pole solenoid is equipped with a current drive (ESCON 50/5, MAXON) and switching power supply (LRS-350-48, MEANWELL), which can generate a magnetic field gradient of 0.1 T/m with unit current input. The observation equipment consists of a microscopic camera (MD028MU-SY, XIMEA) and a telephoto lens (FA2502D, CHIOPT), which can transmit and record 16 fps grayscale image signals. The driven system can generate programmable uniform rotating magnetic fields and oscillating magnetic fields within the spherical workspace (*r* = 100 mm).

To verify the accuracy of the magnetic field, we measured the actual magnetic field intensity and gradient while working with a single pair of coils and compared them to their theoretical values. When a unit current was passed, measurements were taken point by point. The center of the axis was designated as the origin, with a sampling point interval of 10 mm, an axial range of −40 to 40 mm, and a radial range of 0 to 40 mm. For the axis of the coil and its parallel lines, the magnetic field and gradient distributions in the middle section were U-shaped, which is in general agreement with theoretical calculations. The central point on the axis corresponds to the minimum point and has a magnetic field of 10.71 mT and a gradient of 0.41 T/m per unit input current. For distances less than 10 mm away from the center point, the error is no more than 5%. In the measuring plane, the center point represents a saddle point, where the field intensity and gradient decrease as the measured point moves outward radially. When the radial distance is less than the core radius, no significant difference exists between the axis and the parallel line. To summarize, the magnetic field and its gradient exhibit good uniformity within a range of 20 to 30 mm in proximity to the central point (see Fig. 5B and C).

### Verification of reversible deformation of the flexible miniature robot

**Fig. 6.**
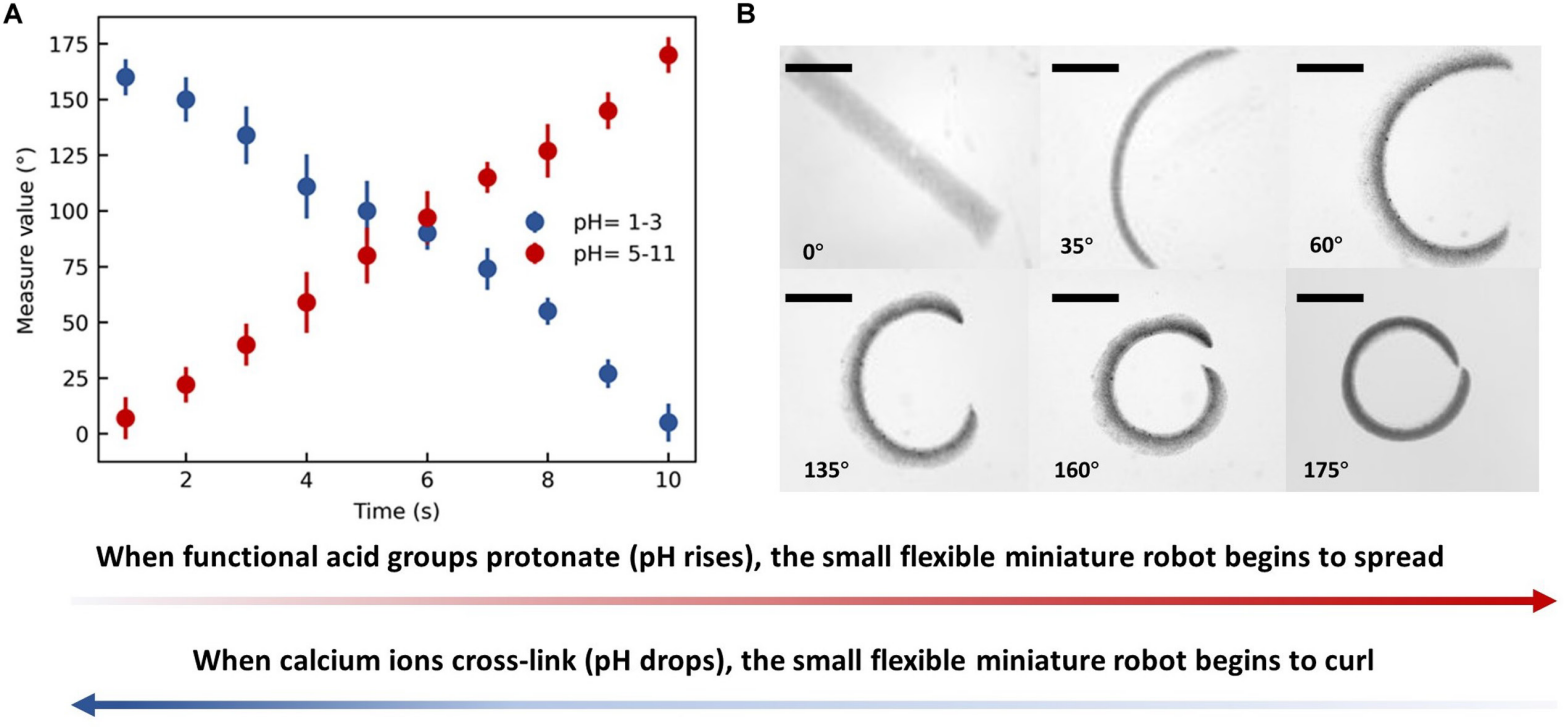
Deformation diagram of the flexible miniature robot. (A and B) The flexible miniature robot shrinks and deforms as pH decreases. The flexible miniature robot spreads as pH rises. The scale bar is 250 μm.

To assess the deformation capabilities of the flexible miniature robot, a series of deformation tests were conducted in this study. When the pH of the environment decreases, the flexible miniature robot undergoes spontaneous contraction, transitioning from a bar shape to a circular shape. As depicted in Fig. 6A to C, the 3 stages of the contraction deformation process are illustrated. The changes in deformation angle (*θ*) and deformation radius (*R*) are clearly visible. As pH levels increase and the environment transitions from acidic to alkaline, the robot expands from its contracted state, transitioning from a ball shape to a bar shape, as illustrated in Fig. 6D to F.

### Multimodal locomotion and velocity characteristics

**Fig. 7.**
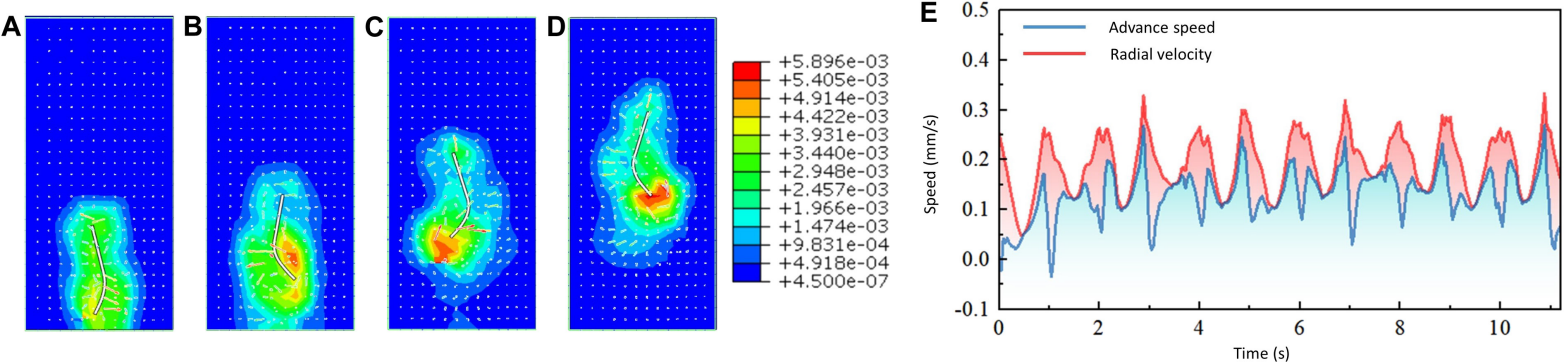
The pendulum motion simulation. (A to D) Motion top view. (E) The diagram of the motion velocity curve.

**Fig. 8.**
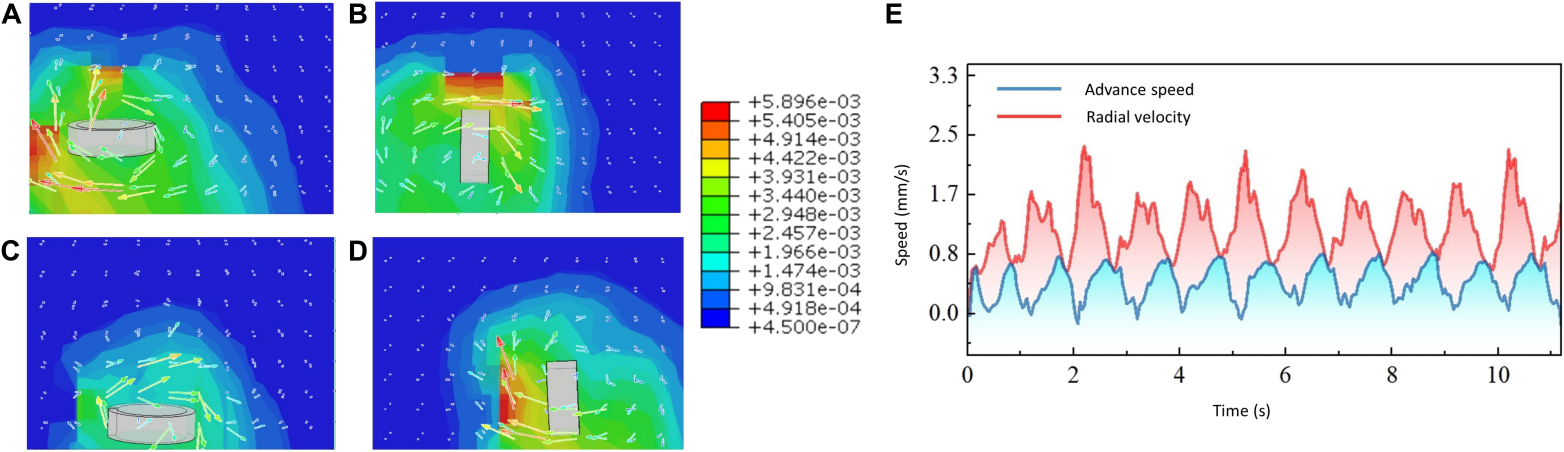
The rolling motion simulation. (A to D) Motion side view. (E) The diagram of the velocity.

In this study, the 2 modes of motion exhibited by the flexible miniature robot are demonstrated: the pendulum motion of the bar-shaped robot and the rolling motion of the ball-shaped robot. Based on the finite element analysis method, we established a fluid–structure coupling simulation model to explain the movement phenomenon and state of the robot in a liquid environment. In our simulation, the liquid medium is modeled as an incompressible, stable laminar flow with an initial velocity of 0. The liquid we chose was pure water (the viscosity is 1 mPa·s) and the temperature was set at a constant room temperature. The fluid domain is 10 times larger than the flexible miniature robot, which reduces the influence of boundary conditions. The flexible miniature robot is assumed to be neutrally buoyant, so its buoyancy and gravity are not taken into account. Figures 7 and 8 show the simulation results of the 2 motion forms, including the velocity program and environmental velocity cloud map, and motion velocity curve.

**Fig. 9.**
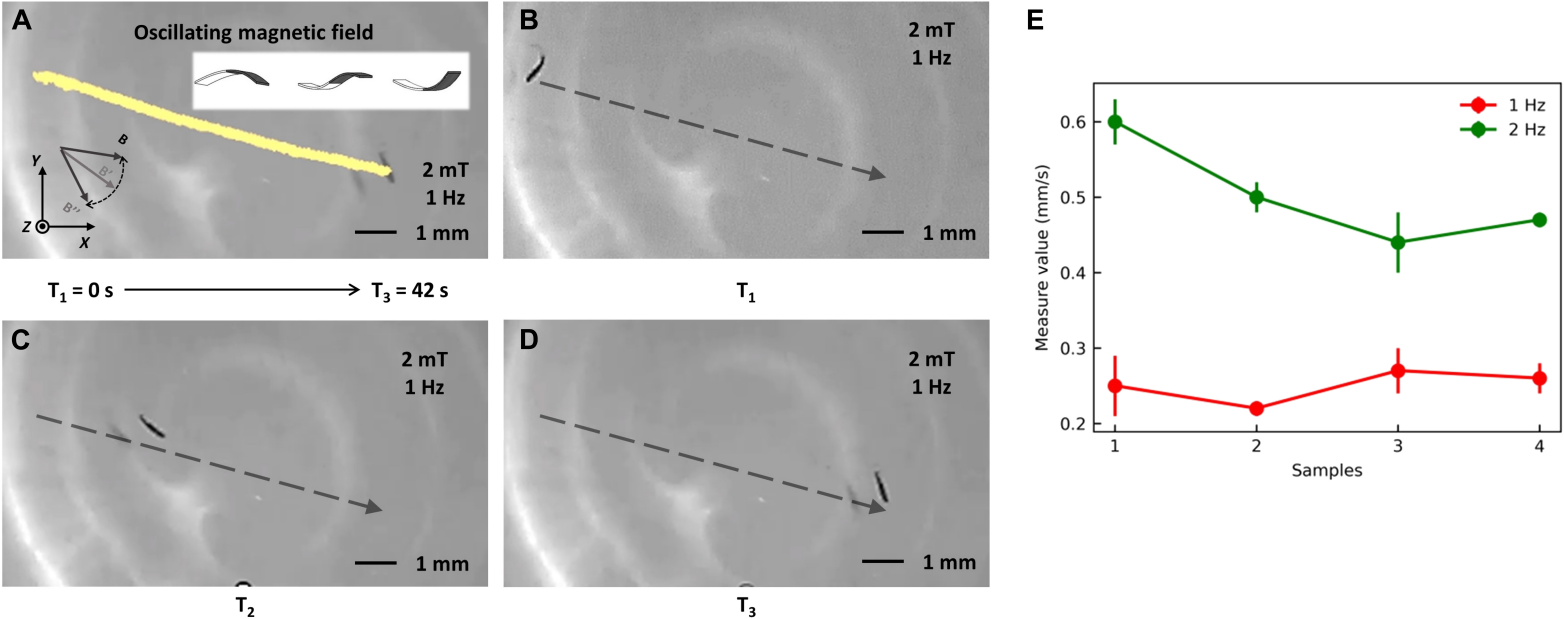
The pendulum motion experiment. (A) The diagram of the actual trajectory of the flexible miniature robot. The yellow line represents the tracing result. (B to D) The flexible miniature robot movement results at different times. The black arrow shows the direction of motion. (E) The diagram of the velocity at different frequencies. The scale bar is 1mm.

**Fig. 10.**
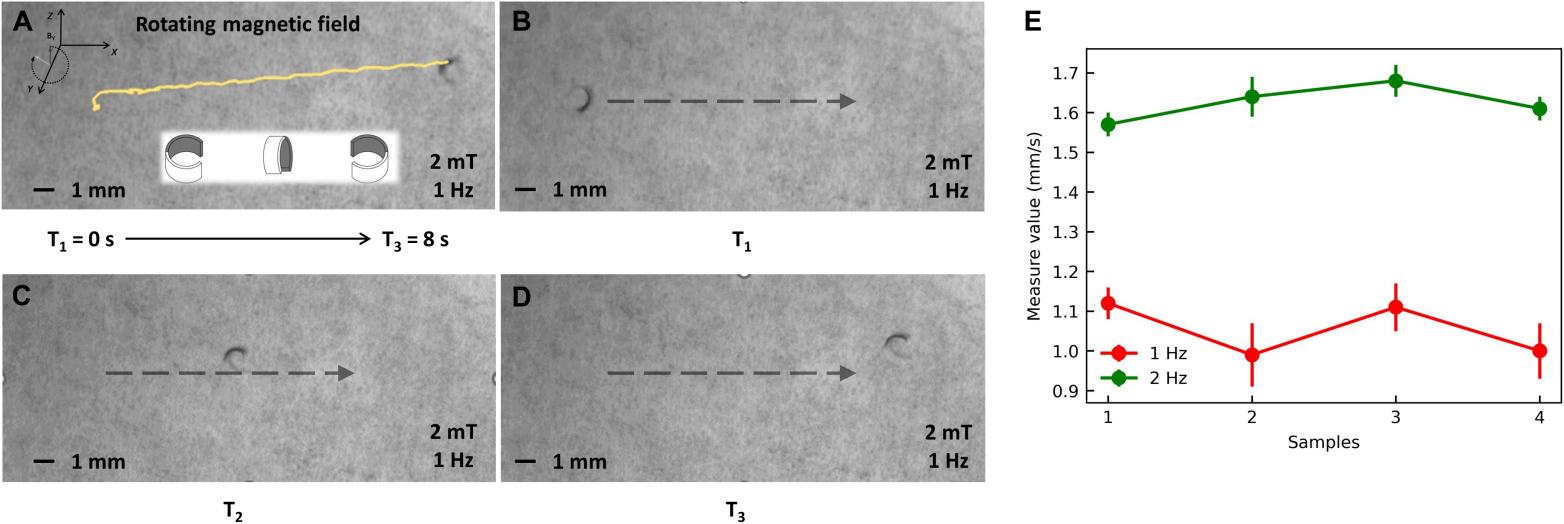
The rolling motion experiment. (A) The diagram of the actual trajectory of the flexible miniature robot. The yellow line represents the tracing result. (B to D) The flexible miniature robot movement results at different times. The black arrow shows the direction of motion. (E) The diagram of the velocity at different frequencies. The scale bar is 1 mm.

In the pendulum mode motion experiment, we tested the motion velocity of a flexible miniature robot at driving frequencies of 1 Hz and 2 Hz. The experimental results are shown in Fig. 9. In an open environment when the driving frequency is 1 Hz, the robot speed is approximately 0.23 mm/s. When the driving frequency increases by 1 Hz, the speed increases by approximately 0.25 mm/s. In the rolling motion experiment, we performed the same operation and found that the flexible miniature robot exhibited similar motion rate characteristics, as shown in Fig. 10. The speed of the rolling motion is significantly higher than that of the swinging motion. At a driving frequency of 1 Hz, the speed is approximately 1.07 mm/s, and a 1-Hz increase in driving frequency results in a 0.5-mm/s increase in speed. To establish a more rigorous correlation between the frequency of the driving magnetic field and the resulting motion speed of our robot, we conducted additional experiments. Our findings support the previous observation that as the external driving magnetic field frequency initially increases, the robot’s motion speed similarly rises until reaching a critical threshold. Beyond this point, however, the robot becomes unable to maintain synchronous response to the magnetic field changes, resulting in a subsequent decline in its motion speed.

**Fig. 11.**
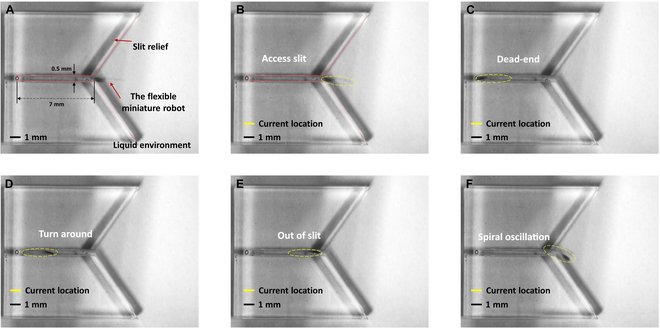
(A) Topographic map. (B) The flexible miniature robot successfully accesses the slit through the implementation of an oscillating magnetic field. (C) The flexible miniature robot arrives at the dead-end. (D) The flexible miniature robot turns around. (E) The flexible miniature robot prepares to leave the slit. (F) The flexible miniature robot gets out of the slit by rotating the magnetic field.

We also demonstrated the mobility of a flexible miniature robot in confined spaces. As depicted in Fig. 11, the flexible miniature robot successfully navigates through a narrow slit, showcasing the entire process, which includes entering, reaching the end, turning in place, leaving, and rotating away. The dimensions of the slit are approximately 7 mm in length and 0.5 mm in width, as depicted in Fig. 11A. Initially, the robot enters the slit with a reduced swing amplitude and an increased swing frequency, as shown in Fig. 11B. Subsequently, the robot reaches the end of the slit and encounters an impediment, unable to proceed further, as illustrated in Fig. 11C. To overcome this obstacle, the robot utilizes its flexible body by retracting and rotating in place, as depicted in Fig. 11D. As a specific segment of the robot’s body exits the slit, it switches to a larger swing amplitude with the objective of swiftly escaping the slit, as demonstrated in Fig. 11F. The entire movement can be seen in Movie S1.

## Discussion

In this study, we developed a flexible miniature robot with a variable form that can achieve multimodal movement and adapt to complex terrain environments. The flexible miniature is constructed using electrodeposition of alginate saline gel, featuring dimensions in a scale of 100:5:2, representing the major axis length, minor axis length, and thickness, respectively. The flexible miniature robot can spontaneously deform in response to changes in the ion concentration of its environment. Experimental results demonstrate that the designed flexible miniature robot can undergo reversible deformation and assume a variety of forms. The maximum deformation angle achieved is 180°. We have designed an oscillating magnetic field to induce forward swaying when the flexible miniature robot is in its bar state. Similarly, when the flexible miniature robot is in its spherical state, we have implemented a rotating magnetic field to facilitate forward rolling. This highly adaptable flexible miniature robot is capable of undergoing spontaneous and reversible deformation in response to environmental changes. Its potential applications include targeted in vivo medical treatments within the digestive system. Nonetheless, several unresolved technical challenges demand further scrutiny in our research. The intricate physiological environment within living organisms necessitates enhanced intelligence of the robot, encompassing a broader spectrum of stimulus–response capabilities and more precise motion control, among other aspects. In future endeavors, our focus will extend to investigating robots capable of responding to multiple stimuli, thereby adapting to diverse complex scenarios. Additionally, we aspire to achieve closed-loop control over the robot’s movement to enable precise operations.

## Data Availability

The data are freely available upon request.
